# Behavior of Aberrant Chromosome Configurations in *Drosophila melanogaster* Female Meiosis I

**DOI:** 10.1534/g3.114.014316

**Published:** 2014-12-09

**Authors:** William D. Gilliland, Eileen M. Colwell, Fiona M. Lane, Ashley A. Snouffer

**Affiliations:** * Department of Biological Sciences, DePaul University, Chicago, Illinois 60614-3207; † Department of Genetics, University of Georgia, Athens, Athens, Georgia 30602-2607

**Keywords:** aneuploidy, chromosome segregation, compound chromosomes, heterochromatin

## Abstract

One essential role of the first meiotic division is to reduce chromosome number by half. Although this is normally accomplished by segregating homologous chromosomes from each other, it is possible for a genome to have one or more chromosomes that lack a homolog (such as compound chromosomes), or have chromosomes with multiple potential homologs (such as in *XXY* females). These configurations complete meiosis but engage in unusual segregation patterns. In *Drosophila melanogaster* females carrying two compound chromosomes, the compounds can accurately segregate from each other, a process known as heterologous segregation. Similarly, in *XXY* females, when the *X* chromosomes fail to cross over, they often undergo secondary nondisjunction, where both *Xs* segregate away from the *Y*. Although both of these processes have been known for decades, the orientation mechanisms involved are poorly understood. Taking advantage of the recent discovery of chromosome congression in female meiosis I, we have examined a number of different aberrant chromosome configurations. We show that these genotypes complete congression normally, with their chromosomes bioriented at metaphase I arrest at the same rates that they segregate, indicating that orientation must be established during prometaphase I before congression. We also show that monovalent chromosomes can move out on the prometaphase I spindle, but the dot *4* chromosomes appear required for this movement. Finally, we show that, similar to achiasmate chromosomes, heterologous chromosomes can be connected by chromatin threads, suggesting a mechanism for how heterochromatic homology establishes these unusual biorientation patterns.

One vital task of the first meiotic division is to halve chromosome number to produce haploid gametes. This reduction usually is accomplished by pairing between homologous pairs of chromosomes, followed by crossing-over to lock homologs together until anaphase I. Even the sex chromosomes in heterogametic individuals (males in *XY* species such as mammals and flies, and females in *WZ* species such as birds and butterflies) are not really an exception to this, because they behave like homologous chromosomes. In male mammals the pseudo-autosomal region allows regular crossing-over to occur on the sex chromosomes (Cooke *et al.* 1985) whereas in male *D. melanogaster* the spacer elements within ribosomal DNA arrays located on the *X* and *Y* pair these chromosomes without crossing-over (McKee *et al.* 1992). However, it is possible for chromosomes to lack sequence homologs. For example, compound chromosomes (single molecules of DNA that carry the euploid gene complement of two full chromosomes) can be created, as first reported for the *Drosophila melanogaster X* by Morgan (1922). This results in gametes carrying either 0 or 2 copies of genes found on that chromosome and leads to lethal aneuploidy when mated to individuals carrying normal unattached chromosomes, a feature that has been investigated for controlling pest populations (Gould and Schliekelman 2004). Although a single such chromosome segregates randomly to either pole at meiosis I, there are several ways two different compound chromosomes could segregate. If segregation was independent, both chromosomes would be distributed at random, with a quarter of gametes containing both chromosomes, a quarter containing neither, and two quarters with a single chromosome each.

Conversely, if segregation was not independent, an excess of gametes where the two chromosomes segregated away from each other (despite being heterologous, instead of homologous, chromosomes) is predicted, enriching for gametes carrying only one of the two chromosomes compared to gametes containing both or neither. This heterologous segregation (HS) takes place in *D. melanogaster* females, as two such chromosomes move to opposite poles at high frequency. For example, 97% of eggs from females carrying both attached-*X* and attached-*4* chromosomes will receive only a single compound (Grell 1963), whereas 99% of eggs from females with compound left and right arms of chromosome *2 (C(2L)/Ø*; *C(2R)/Ø)* contain a single compound (Grell 1970). However, not all combinations of heterologs are as efficient at cosegregating. This was most clearly shown in a study of a graded series of *Dp(1;f)* chromosomes on the segregation of *X* and *4* chromosomes (Hawley *et al.* 1993). As duplications incorporated larger amounts of *X* and *4* heterochromatin, they became more effective at inducing *Dp<=>XX* and *Dp<=>44* segregation events. Additionally, sufficient overlap in the centromeric heterochromatin was necessary for accurate segregation in a study of minichromosome derivatives (Karpen *et al.* 1996). Therefore, it is clear that there must be a mechanism in female meiosis that can biorient heterologous chromosomes before their eventual segregation and that this process requires heterochromatic homology.

A conceptually similar phenomenon also is observed in *XXY* females. While recombinant *X* chromosome homologs segregate properly from each other, if the *Xs* fail to cross over (which occurs spontaneously in 5–10% of meioses, and in 100% of meioses in females heterozygous for rearranged balancer chromosomes) the two *X*s will frequently segregate away from the *Y* (Grell 1962b). This phenomenon was first identified by Bridges (1916), who termed it secondary nondisjunction, as a primary nondisjunction event is necessary to initially produce the *XXY* females. Subsequent confocal work showed that the sex chromosomes in *XXY* females could be found out on the prometaphase I spindle (meaning they are positioned between the chromosomes located at the metaphase plate and the spindle pole on one side of the spindle) at rates very near to their segregation rate (Xiang and Hawley 2006). Although the *X* and *Y* chromosomes are both sex chromosomes and therefore segregate as homologs in males, female meiosis normally lacks a *Y*. The ability of a *Y* chromosome to disrupt the normal segregation of the homologous *X* chromosome pair, but only when the *X* chromosomes fail to recombine, suggests that secondary nondisjunction may be related to HS, although it is unknown if the same mechanism drives both processes.

Based on a number of classical genetic studies of flies bearing chromosome rearrangements, Grell (1962a) proposed that HS occurs in a second round of pairing, after exchange-mediated chromosome segregation. This hypothesis was evaluated in several early confocal cytology studies of female meiosis. Although compound chromosomes could be seen out on the spindle, with the two heterologs associated with opposite poles and therefore bioriented (Theurkauf and Hawley 1992; Hawley *et al.* 1993; Dernburg *et al.* 1996), they found that both homologous and heterologous chromosomes proceeded through prometaphase I at the same time. Based on these data, Hawley and Theurkauf (1993) concluded that all chromosomes moved out on the spindle, and that no such second round of biorientation existed.

These previous cytological studies were interpreted using a model in which the nonexchange chromosomes were out on the spindle at metaphase I arrest, separated from the exchange chromosome mass (Theurkauf and Hawley 1992). However, several recent discoveries have changed our understanding of prometaphase I. First, the configuration with the chromosomes out on the spindle is actually the midpoint of prometaphase I, and those chromosomes must subsequently rejoin the exchange chromosomes, congressing to a single mass at metaphase I arrest (Gilliland *et al.* 2009). Second, rather than moving out toward the spindle poles independently, nonexchange chromosomes instead remain connected by chromatin threads, allowing a separate-and-rejoin cycle that establishes co-orientation (Hughes *et al.* 2009). Although the content of these chromatin threads is not fully understood, they appear to contain heterochromatin, based on threads being highlighted by both heterochromatin-specific fluorescence *in situ* hybridization (FISH) probes and a phospho-specific histone antibody (Hughes *et al.* 2011). Finally, it was shown that the rate at which homologs are co-oriented at metaphase I arrest predicts the segregation rate observed in the progeny (Gillies *et al.* 2013).

These recent advances in our understanding of prometaphase I provide an opportunity to reexamine the segregation of aberrant chromosome configurations. To this end, we have examined prometaphase I and metaphase I arrest in females carrying one or more compound chromosomes as well as *XXY* females. We show in fixed oocytes that all of these genotypes complete congression to a single chromosome mass at high rates. In females carrying a monovalent compound chromosome, we show that a compound *X* or *2* can be found out on the spindle during prometaphase I but that in flies carrying a compound *4*, this movement is greatly curtailed, even when recombination on the *X* was blocked. This finding suggests that the dot *4s* may be required for this normal chromosome movement. We also show that at metaphase I arrest, heterologous biorientations occur at similar rates to meiotic segregation rates inferred from progeny counts. Finally, we show that heterologous chromosomes can be connected by chromatin tethers, providing a possible mechanism for how heterochromatic homology causes heterologous biorientation. Together, these results provide insights into HS as well as the normal role of the small *4* chromosomes.

## Materials and Methods

### Starting stocks

The following stocks were from the Bloomington Drosophila Stock Center: *Dp(1;Y)B^S^ y^+^ Y / +; C(2)EN, bw^1^ sp^1^ / Ø* (BL-1111). *C(2L)RM-P1, b^1^/Ø; C(2R)RM-P4, px^1^* (BL-713). *C(3L)RM-P3, kni^ri-1^/Ø; C(3R)RM-P3* (BL-718). *In(1)dl-49, v^Of^ f^1^* (BL-779). The remaining stocks were from our stock collection: Oregon-R, *y^2^ cv^1^ v^1^ f^1^, y^1^ w^1^/y^+^Y; sv^spa-pol^, C(1)RM, y^1^ v^1^/Ø; C(4)RM, ci^1^ ey^R^/Ø* females x *C(1;Y^S^), v^1^ f^1^ B^1^/Ø; C(4)RM, ci^1^ ey^R^/Ø* males, and *FM7w, y w B/y^+^Y; sv^spa-pol^.* Allele numbers are hereafter omitted. These stocks were used to create two additional stocks *(y w f/y^+^Y; sv^spa-pol^* and *y In(1)dl-49, v f/y^+^Y; sv^spa-pol^)* using standard recombination and segregation methods. Additionally, *C(1)RM, y v/Ø; C(4)RM, ci ey^R^* females were crossed to *y w f / y^+^Y; sv^spa-pol^* males to isolate the two compounds into separate *C(1)RM, y v / Ø; sv^spa-pol^* and *y w f; C(4)RM, ci ey^R^ / Ø* stocks, by the use of secondary nondisjunction in the cross.

To produce *XXY* females, stocks bearing a marked *y^+^Y* were grown until a *y^+^* female was produced by spontaneous *X* nondisjunction. This female was propagated to produce bottles of *XXY* females by selecting *y*^+^ females each generation, who were mated back to new males from the source stock (to avoid *XYY* males and *XXYY* females.)

To produce *XXY* females with an unmarked *Y*, *y w f / y w f / y^+^Y*; *sv^spa-pol^* virgin females were crossed to *y In(1)dl-49*, *v f/y^+^Y*; *sv^spa-pol^* males, and then *y w f / y In(1)dl-49*, *v f / y^+^Y*; *sv^spa-pol^* female progeny (from *XY*-bearing oocytes, produced by normal *X* segregation) were then crossed to Oregon-R males, and *y w f / y In(1)dl-49*, *v f / Y* female progeny (from *XX*-bearing oocytes, produced by secondary nondisjunction) were collected.

### Genetic segregation assays

Single females were crossed to appropriately marked males that allowed identification of segregation products in yeasted vials, allowed to lay eggs for 5-6 d, and then adults were cleared. Progeny were then scored up through 18 d after vials were set up. For *XXY* females, half of the *XX*- and *Y*-bearing oocytes are expected to die from receiving the wrong sperm genotype, so surviving nondisjunctional (NDJ) progeny were doubled to compensate (Zeng *et al.* 2010). For some crosses, one of the two exceptional classes was haplo-*4* minute with poor viability, so the number of the other class was doubled again to compensate.

### Meiotic stage enrichment

Enrichment of oocytes in either mid-prometaphase I or metaphase I arrest was done by manipulation of female age and mating status (Gilliland *et al.* 2009). In summary, when prometaphase I oocytes were desired, newly eclosed virgin females were aged for 42−48 hr in vials with yeast and males. These females are still increasing their rate of egg production and therefore have many oocytes in mid-prometaphase I, whereas mature oocytes are laid and cleared from the ovary. For preps where metaphase I arrested oocytes were desired, newly eclosed virgin females were aged for 4 or 5 d in vials with yeast and no males. These females hold their mature oocytes, and so contain many oocytes at metaphase I arrest.

### Ovary dissection and fixation

Ovaries were hand-dissected in 1x Robb’s media + 1% bovine serum albumin, then fixed in 1×WHOoPASS + 8% paraformaldehyde as described (Gillies *et al.* 2013). For FISH preps, the two fixative components were preheated to 39°, combined immediately before application to the sample, and fixed on a stationary heating block at 39°. All other preps were fixed at room temperature on a nutator.

### 4′,6-Diamidino-2-phenylindole (DAPI)-only preps

Fixed ovaries were washed in phosphate-buffered saline with Triton X-100 (PBST), ovarioles were separated via rapid pipetting through a p1000, washed 3× in PBST for 15 min, then stained with 1× DAPI for 6 min. Oocytes were then washed in PBST for 3 brief washes and 2 15-minute washes, then mounted in SlowFade Gold (Invitrogen).

### FISH preps

Fixed oocytes were washed in 2× saline-sodium citrate buffer with Tween 20 and FISH was performed as described (Gilliland *et al.* 2009), using 92° melting and 32° annealing temperatures.

The chromosome-specific FISH probes used were as follows: *X*, TTT-TCC-AAA-TTT-CGG-TCA-TCA-AAT-AAT-CAT (Ferree and Barbash 2009); *Y*, (AATAC)_6_, *2L* (AATAG)_6_, *2L-3L* (AATAACATAG)_3_, *2R* (AACAC)_6_, and *4* (AATAT)_6_ (Dernburg 2000). All probes were synthesized with fluorescent labels by IDTDNA.com or were a generous gift from the lab of R. Scott Hawley.

### Antibody preps

Fixed oocytes were washed in PBST, dechorionated by rolling between frosted glass slides, washed 3×, blocked for 1 hr in PBST-normal goat serum (NGS), and then hybridized overnight in PBST-NGS at 4° to rat anti-tubulin (Serotec MCA786, 1:250) and rabbit anti-pH 3S10 (Millipore #06-570, 1:500) primary antibodies. Oocytes were given three brief and one 15-min PBST wash, blocked for 1 hr in PBST-NGS, then hybridized for either 4−6 hr at room temperature, or overnight at 4°, to goat anti-rat IgG with conjugated Alexa 647 fluorophore and goat anti-rabbit IgG with conjugated Alexa 564 (both Invitrogen, 1:250) secondary antibodies. DAPI staining, washing, and mounting were then done as described for DAPI-only preps previously.

### Microscopy and deconvolution

Slides were visualized on a Leica SPE-II confocal using LAS AF software (www.leica.com). Where oocyte counting was needed, an image of the entire slide was taken with a dissecting microscope and used to guide oocyte selection. This ensured all oocytes in an area could be scored without being missed or double-counted. Oocytes were marked at low magnification using the Mark and Find panel using DAPI visualization and without examination of chromosome orientation, then scored at 63×. Presented images were deconvolved using Huygens Essential (www.svi.nl) using an estimated PSF with default parameters, except mounting media refractive index was set to 1.42 per manufacturer. Channels were separated and projected individually as grayscale TIFFs, then combined to produce color images using channels in Photoshop.

## Results

### Aberrant configurations complete congression

We first asked whether these aberrant chromosomal configurations, such as monovalent compounds, multiple compounds, or *XXY* females, completed congression normally. We examined fixed oocytes from virgin females that were aged 4−5 days post eclosion, which enriches for oocytes at metaphase I arrest (Gilliland *et al.* 2009). For each genotype examined in more detail below, we found the mature oocytes almost exclusively had all their chromosomes congressed into a single mass; most exceptions were oocytes that were fixed while still in prometaphase I, as based on dorsal appendage appearance. This demonstrates that these large chromosomal aberrations complete congression normally.

### Chromosomes without pairing partners

**Figure 1:**
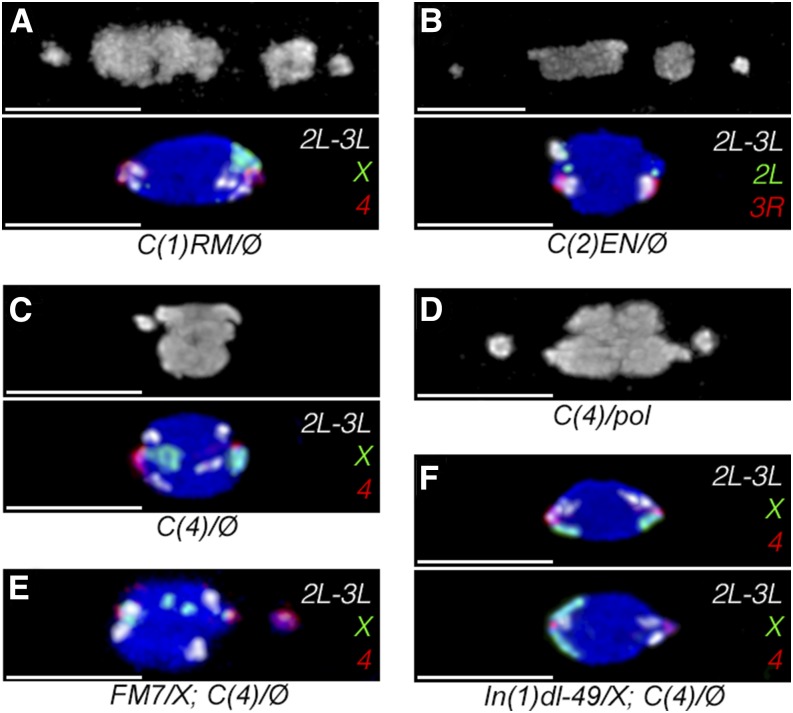
Chromosomes without pairing partners. All spindles are oriented horizontally (scale bars = 5 µm) and are stained with DAPI (gray/blue). (A) Top panel shows prometaphase I in a *C(1)RM*, *y v /Ø*; *sv^spa-pol^* oocyte, with the large *C(1)* out on the right spindle arm. Bottom panel shows metaphase I arrest in the same genotype, using *2L-3L* (white), *X* (green), and *4* (red) probes, indicating the *4s* are co-oriented with the *C(1)* oriented to the right pole. (B) Top panel shows prometaphase I in a *C(2)EN*, *bw sp/Ø* oocyte, with the large *C(2)* on the right arm of the spindle, between the normal *4* and the exchange *X* and *3*. Bottom panel shows metaphase I arrest in the same genotype, using 2L-3L (white), 2L (green), and 3R (red) probes. Note the *C(2)* on the left is highlighted by both white and green probes, while small spots of 2L probe are found on the co-oriented *4s*. (C) Top panel shows the only oocyte from 2-d mated *y w f*; *C(4)RM*, *ci ey^R^/Ø* females where the *C(4)* was found out on the spindle. The distance the *C(4)* has moved is quite small. Bottom panel shows metaphase I in the same genotype, with the same probes as in A, and the *C(4)* oriented left. Note the *X* also has a small spot of *4* probe. (D) The *C(4)* chromosome is competent to move out on the spindle, as can be seen in this *C(4)/ sv^spa-pol^* oocyte. (E) In *FM7/y w f*; *C(4)/Ø* oocytes (labeled with same probes as (A), chromosome movements occur less often, but the *C(4)* can be found out on the spindle. Note both the normal *X* (left) and *FM7* (right) chromosomes hybridize *4* probe near their centromeres, while the *FM7* chromosome has most of the *X* probe moved distally by a large inversion, found in two spots near the center of the chromosome mass. (F) Top panel shows normal *XC(4)<=>X* coorientation in a *y w f / y In(1)dl-49*, *v f*; *C(4)/Ø* oocyte, while bottom panel shows heterologous *XX<=>C(4)* orientation.

We first examined females carrying monovalent compound chromosomes, which lack a homologous pairing partner entirely. In 2-d mated *C(1)RM*, *y v/Ø*; *sv^spa-pol^* females, we found 56% of oocytes had chromosomes out on the prometaphase I spindle, with the unpaired *C(1)* chromosome out in 32% of oocytes (Table 1). The *4* chromosomes were found properly positioned on opposite sides of the spindle, and closer to the poles than the *C(1)* (Figure 1A, top). In aged virgins, we found 93% of oocytes with chromosomes in a single mass (Figure 1A, bottom). Of the exceptions, five were still in prometaphase with the *4s* properly co-oriented (suggesting those oocytes had not yet completed congression), two had the major autosomes split into separate compact masses, and two were in a heterologous *C(1)<=>44* configuration (2% HS). These cytological results are consistent with the rate of HS measured genetically in this stock (Table 1).

**Table 1:** Chromosomes without pairing partners

Genotype	Cytological Data							Genetic Data			
	2-d Mated Females			4-d Virgin Females				0-to 18-d Progeny Counts			
	Single Mass	1+ Out	C(n) Out	Single Mass	1+ Out	HS Config.	Cytological HS Rate	Normal Progeny	HS Progeny	Genetic HS Rate	Notes
*C(1)RM*, *y v / Ø*; *sv^spa-pol^*	32	40	23	112	9	2	2%	182	1	2%	*^a^*,*^b^*
*C(2)EN*, *bw sp / Ø*	29	60	28	57	8	2	3%	n.d.	n.d.	n.d.	*^c^*
*y w f*; *C(4)RM*, *ci ey^R^ / Ø*	72	2	2	60	0	2	3%	1136	0	0%	*^a^*,*^d^*
*y w f / y In(1)dl-49*, *v f*; *C(4)RM*, *ci ey^R^ / Ø*	54	7	7	78	2	5	6%	626	9	5.4%	*^a^*,*^e^*
*y w f / FM7*, *y w B*; *C(4)RM*, *ci ey^R^ / Ø*	60	8	8	70	2	5	7%	1232	25	7.5%	*^a^*,*^f^*

Cytological and genetic data for females carrying monovalent compound chromosomes. For each row, cytological data are the number of oocytes scored in each configuration. *Single Mass* and *1+ Out* are mutually exclusive columns, whereas *C(n) Out* is the subset of the *1+ Out* oocytes, where the compound chromosome was out on the spindle, and *HS Config*. is the subset of oocytes where other chromosomes were bioriented away from the homolog. HS, heterologous segregation; n.d., not determined.

a Because only one of the two products of HS are viable, and 50% of those are expected to die from the wrong sperm genotype, the number of HS progeny is quadrupled to estimate the genetic HS rate. No such correction is needed to cytological data.

b HS configurations were *C(1)<=>44*. The genetic data are the sum of two crosses to different male genotypes, *C(1;Y^S^)*, *v f B/Ø*; *C(4)RM*, *ci ey^R^/Ø* (63 normal, 1 HS) and *y w f/y^+^Y*; *C(4)RM*, *ci ey^R^/Ø* (119 normal, 0 HS). The low fecundity in these crosses (~4 progeny/female) is not understood.

c HS configurations were *44<=>C(2)*; *XX<=>C(2)* also was possible but not observed. Genetic segregation data could not be collected because of inviability.

d The observed HS configurations were *C(4)<=>AA*, which would not be recoverable in the progeny; *C(4)<=>XX* was possible but not observed. The genetic data are the sum of two crosses to different male genotypes, *C(1;Y^S^)*, *v f B/Ø*; *sv^spa-pol^* (483 normal, 0 HS) and *FM7*, *y w B/y^+^Y*; *sv^spa-pol^* (645 normal, 0 HS, and 6 progeny from male NDJ).

e HS configurations were *XX<=>C(4)*. Females were crossed to *y w / y+Y*; *sv^spa-pol^*males. A single recombinant male progeny (*w^+^ v^+^ f^–^)* was also recovered.

f HS configurations were *XX<=>C(4)*. Females were crossed to *y w / y^+^Y*; *sv^spa-pol^*males. A single *y^+^ B^+/−^ f^+^* female also was recovered; testcrossing showed the genotype was *FM7/ywf/y^+^Y*; *C(4)/ sv^spa-pol^* and must have come from a nonheterologous *XX;C(4)<=>Ø* oocyte. This fly was not included in the HS rate calculation.

Similar results were found in *C(2)EN*, *bw sp / Ø* females, with at least one chromosome out on the spindle in 67% of oocytes, and the *C(2)* chromosome out in 31% of oocytes (Table 1). In all but two figures, the *4* was closer to the pole than the *C(2)* on that spindle arm (Figure 1B, top), with the two exceptions appearing to have been fixed while in transient join-and-reorient configurations (Hughes *et al.* 2009) instead of stable co-orientations. When we examined 4-d virgin *C(2)EN*, *bw sp / Ø* females, we found chromosomes in a single mass in 88% of oocytes, with the exceptions appearing to still be in prometaphase I. The *4s* were properly co-oriented in 97% of oocytes (Figure 1B, bottom; Table 1), with the exceptions in a heterologous *44<=>C(2)* configuration, suggesting the compound autosome is only mildly disrupting the segregation of other chromosomes. Therefore, it appears that a monovalent *C(1)* or *C(2)* can still move out onto the spindle, even though they lack a pairing partner, and these chromosomes complete congression at high rates without greatly disrupting the other chromosomes.

In contrast, when we examined oocytes in 2-d mated *y w f*; *C(4)RM*, *ci ey^R^/Ø* females, we found only 3% of oocytes with any chromosomes out on the spindle. Of the two exceptions, one had the *4* barely separated from the rest (Figure 1C, top), whereas the other appeared to be in anaphase. In aged virgin females, the chromosomes were always in a single mass, and properly co-oriented 97% of the time (Figure 1C, bottom), with both exceptions having a *C(4)<=>AA* autosomal malorientation, which would be lethal in the progeny. This cytological data are consistent with the complete lack of HS observed in the genetic data from this stock (Table 1).

That the *C(4)* could be found adjacent to the other chromosomes, but with very little space separating it from the other chromosomes, raised the possibility that the *C(4)* could not move out onto the spindle at all. We generated *ywf / yw*; *C(4)RM*, *ci ey^R^/ sv^spa-pol^* females, which are viable and fertile and should have pairing between the *sv^spa-pol^* and *C(4)* chromosomes. In 2-d mated females, we found chromosomes out on the spindle in 37 of 80 oocytes (46%), and could find both *4* and *C(4)* well separated (Figure 1D), indicating that although the *C(4)* could move out on the spindle, it did not appear to do so as a monovalent.

This raised a question about the role that the small *4* chromosome normally plays in prometaphase I chromosome movements. One possibility is the presence of two normal *4s* means that *D. melanogaster* female meiosis always contains at least one pair of homologous nonexchange chromosomes, and this is what causes chromosome movement. Under this model, any nonexchange chromosome should suffice for movement to occur. Alternatively, the *4s* themselves could be required for prometaphase I chromosome movement, and without a pair of free *4s* the other chromosomes are unable to move out on the spindle. Under this model, the presence of the two *4s* would be necessary to organize the chromatin threads, guiding the movement of other chromosomes. Because the *y w f*; *C(4)RM*, *ci ey^R^ / Ø* females lack both free *4s* and nonexchange homologous pairs, no chromosome movement would be expected under either model. However, if another nonexchange chromosome were introduced, such as by preventing *X* chromosome crossing over, the two models make different predictions. If any nonexchange chromosome suffices to induce movement, then blocking recombination on the *X* should cause the *X* chromosomes to be readily found out on the spindle, with the monovalent *C(4)* farther from the pole than the nonexchange chromosomes [analogously to the monovalent *C(1)* or *C(2)* being positioned closer to the spindle midzone than the normal *4s*]. Conversely, if two *4s* are essential for prometaphase I chromosome movements, then preventing *X* recombination should not lead to chromosomes being out on the spindle, and the *C(4)* should still be closer to the spindle pole than the nearest *X*.

To test this, we generated both *y w f / y In(1)dl-49*, *v f*; *C(4)RM*, *ci ey^R^ / Ø* and *y w f / FM7*, *y w B*; *C(4)RM*, *ci ey^R^ / Ø* females and examined chromosome positioning. *FM7* is an *X* chromosome balancer with rearranged heterochromatin that completely blocks crossing-over, whereas *In(1)dl-49* is a large inversion that greatly reduces crossing over while retaining normal *X* heterochromatin. In mated 2-d females of both genotypes, we found ~13% of oocytes with chromosomes out on the spindle (Table 1), an increase from *C(4)/Ø* with exchange *Xs* but well below the ~60% seen when free *4s* were present. Furthermore, in all oocytes where chromosomes were out on the spindle, it was the *C(4)* chromosome that was positioned closest to the spindle pole on that half of the spindle (Figure 1E), although the distance that chromosomes did move out appeared reduced. In aged virgin females of these genotypes, most oocytes were in a single mass and properly co-oriented (Figure 1F, top) whereas heterologous *C(4)<=>XX* biorientations (Figure 1F, bottom) were seen in both genotypes at rates close to the genetic HS rates measured in these stocks (Table 1). Therefore, although a pair of free *4* chromosomes is not necessary for prometaphase chromosome movements, the reduced frequency of movement, and the positioning of the monovalent *C(4)* closest to the spindle pole, suggests the *4* may normally facilitate this process.

### Chromosomes with heterologous pairing partners

We then investigated genotypes with multiple heterologous chromosomes. Although classical genetic analysis had shown that HS were not due to inviability of certain progeny classes (Holm and Chovnick 1975), our cytological approach of measuring biorientation via FISH at metaphase arrest allows direct validation of this inference. Therefore, we did chromosome-specific FISH in aged virgin females to determine chromosome configuration, with the prediction that the rates of chromosome biorientation at metaphase I arrest should be equal to the segregation rates inferred from progeny counts across genotypes.

**Figure 2:**
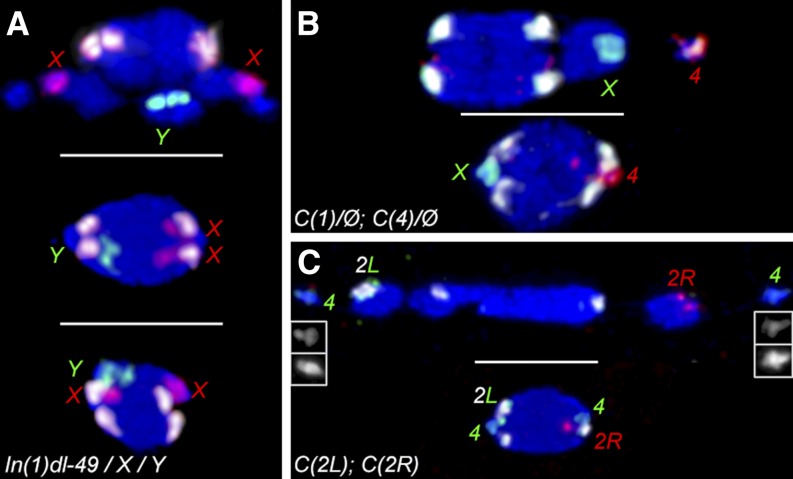
Chromosomes with multiple pairing partners. All spindles are oriented horizontally (scale bars = 5 µm) and stained with DAPI (blue). (A) Chromosomes from three *In(1)dl-49*, *y v f / y w f / Y* oocytes are shown, separated by scale bars, with *X* probe (red), *Y* probe (green), and *2L-3L* probe (white). The top oocyte is still in prometaphase I, with chromosomes out on the spindle showing how all chromosomes are labeled, including the small unlabeled *4s*. The center chromosome mass is at metaphase I arrest and is in a heterologous configuration, with the *Y* oriented to the left pole whereas both *X*s are oriented to the right pole. The bottom chromosome mass is at metaphase arrest in a homologous configuration, with the *X* chromosomes co-oriented. (B) Chromosomes from two *C(1)RM*, *y v / Ø*; *C(4)RM*, *ci ey^R^ / Ø* oocytes, labeled with *X* probe (green), *4* probe (red), and *2L-3L* probe (white). The top oocyte is still in prometaphase I, with both *X* and *4* chromosomes out and on the same side of the spindle. The bottom oocyte is at metaphase I arrest, with the two compound chromosomes bioriented in a heterologous *C(1)<=>C(4)* configuration. (C) Chromosomes from two *C(2L)RM-P1*, *b*; *C(2R)RM-P4*, *px* oocytes, labeled with *2L* probe (green), *2R* probe (red), and *2L-3L* probe (white). The top oocyte is in prometaphase I, with the *4* chromosomes on the outside and the two compound chromosomes separated across the spindle. The top insets show the *4s* labeled with *2L* probe whereas the bottom insets show the *4s* labeled with DAPI. The bottom oocyte is at metaphase I arrest, with the two compound chromosomes in a heterologous *C(2L)<=>C(2R)* configuration. Note that only three white spots are visible, with two on the side with the *C(2L)* chromosome.

We first considered secondary nondisjunction (NDJ) in *XXY* females, where the normal homologous segregation pattern is for the *X* chromosomes to separate from each other, with the *Y* going to one pole at random, while the HS pattern is for the *Y* to segregate to one spindle pole while the two *X* chromosomes go together to the other pole (Figure 2A). When the *X* chromosomes are capable of recombination, only the 5–10% of chromosomes that spontaneously fail to recombine should undergo secondary NDJ. We found only 4% of oocytes in the *Y<=>XX* configuration, close to the genetic segregation rate (Table 2). When the *X* chromosome cannot undergo recombination, either by heterozygosity for an inversion or a balancer chromosome, then the rate of secondary NDJ is much higher (Xiang and Hawley 2006). When we reduced *X* chromosome recombination, we also found comparable proportions of oocytes with the sex chromosomes in heterologous configurations, with either rearranged heterochromatin *(FM7 / X / y^+^Y*, 63%) or normal heterochromatin *(In(1)dl-49 / X / Y*, 67%). These rates were similar to the genetic rates of *XX<=>Y* segregations (Table 2).

**Table 2:** Chromosomes with multiple pairing partners

Genotype	Cytological Data				Genetic Data				
	4-d Virgin Females				0-to 18-d Progeny Counts				
	Normal Orientation	HS Orientation	Other Orientation	% HS Orientation	Normal Segregation	HS Segregation	Other Segregation	% HS Segregation	Notes
*y w / y w / y^+^Y*	52	2	0	4%	2076	40	0	3.7%	*^a^*,*^b^*
*FM7*, *y w B / y w / y^+^Y*	19	33	0	63%	1356	1077	0	61.3%	*^a^*,*^b^*
*In(1)dl-49*, *y v f / y w / Y*	27	56	0	67%	1426	1371	28	65.4%	*^a^*,*^c^*
*C(1)RM*, *y v / Ø*; *C(4)RM*, *ci ey^R^*	0	65	3	96%	0	600	18	97.1%	*^a^*,*^d^*
*C(2L)RM-P1*, *b / Ø*; *C(2R)RM-P4 / Ø*, *px*	0	45	1	98%	0	2479	26	99.0%	*^e^*

Cytological and genetic data for *XXY* females undergoing secondary nondisjunction and females carrying multiple compound chromosomes undergoing HS. *Cytological Data* are the numbers of oocytes scored in each configuration, whereas *Genetic Data* are progeny counts from experimental crosses. HS, heterologous segregation

a The number of HS progeny is doubled to compensate for the expected 50% lethality because of sperm genotype. No such correction is needed for cytological data.

b Genetic data from Xiang and Hawley (2006).

c Experimental females were crossed to *y w f / y^+^Y* males, resulting in 1371 secondary NDJ progeny (696 *y w f / Y* males and 675 *In(1) y v f / y w / y^+^Y* females) and 1426 normal progeny (367 *y w / y^+^Y* males, 350 *In(1) y v f / y^+^Y* males, 352 *y w / y w f* females and 357 *In(1) y v f / y w f* females). *Other Segregation* includes 25 recombinant progeny (6 *y w f / y^+^Y* males, 10 *In(1) y v / y^+^Y* males, 5 *y v / y w f* females and 4 *y w f / y w f* females. The first chromosome listed is the maternally derived recombinant, for a *v-f* map distance of 0.6 cM, indicating ~95% reduction in recombination rate from normal, and 3 progeny from male NDJ.

d The *Other Orientation* oocytes were two *X4<=>Ø* and one *X4<=>AA*. *Genetic Data* from Grell (1963), Table 4.

e The *Other Orientation* oocyte was *C(2L)<=>C(2R)44*. *Genetic Data* from Grell (1970), Table 1.

Next, we examined females carrying multiple compound chromosomes. In these genotypes, the heterologous configuration is for both compounds to biorient toward opposite poles at metaphase I arrest, with the nonheterologous configuration having both compounds pointed to the same pole. In *C(1)RM*, *y v / Ø*; *C(4)RM*, *ci ey^R^ / Ø* females, the two compounds each lack a homolog and segregate from each other at high rates. Using chromosome-specific FISH probes for both compounds (Figure 2B), we found that these chromosomes also were bioriented at metaphase I arrest in 96% of oocytes, similar to the genetic rate (Table 2). Likewise, in *C(2L)RM-P1*, *b / Ø*; *C(2R)RM-P4*, *px / Ø* females, regular heterologous biorientation at metaphase I was seen in 98% of oocytes (Figure 2C), again a rate similar to the genetic data (Table 2). Therefore, we can directly confirm that HS is caused by the two heterologs cosegregating and not due to inviability of certain segregational classes.

### Heterochromatin threads in heterologously segregating chromosomes

**Figure 3:**
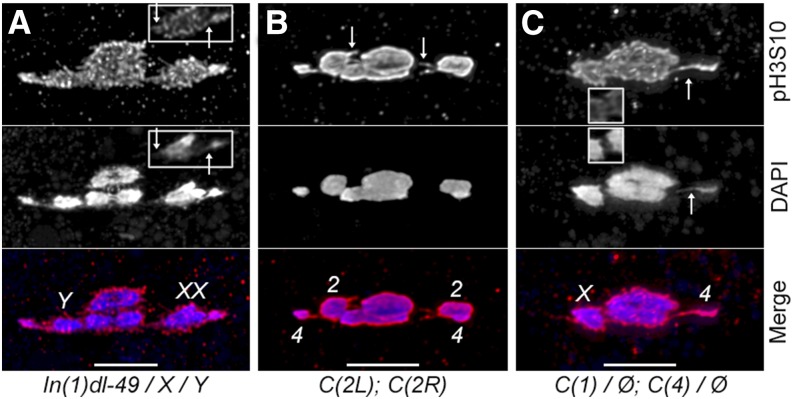
Chromatin threads on heterologously segregating chromosomes. All spindles are oriented horizontally (scale bars = 5 µm) with channels separated for clarity. (A) A prometaphase I oocyte from *In(1)dl-49*, *y v f / y w f / Y* females showing threads coming off the two *X* chromosomes (identified by the brighter DAPI staining of the centric heterochromatin) that are robust enough to see by DAPI as well as *pH 3S10* in a single Z section (insets). Note the thread from the *4* goes along the bottom (up arrow), whereas the threads from the *X* comes from the top (down arrow). (B) A prometaphase I oocyte from *C(2L)RM-P1*, *b*; *C(2R)RM-P4*, *px* shows threads coming from both *C(2)* chromosomes in antibody staining (arrows); threads are not bright enough to see with DAPI. Note that without FISH the *2L* and *2R* cannot be identified from each other, but as all other chromosomes can be identified by DAPI alone the identification of the two compound chromosomes is unambiguous. (C) A prometaphase I oocyte from *C(1)RM*, *v^1^ f^1^ / Ø*; *C(4)RM*, *ci^1^ ey^R^ / Ø* showing a robust thread from the *C(4)* (arrows). Threads from the *C(1)* are obscured in the stack projection but can be clearly seen in a single Z section (insets).

Although these data show that aberrant chromosomes are congressing normally and that heterologous biorientations are established by the time congression finishes, they still do not address the mechanism of how these heterologous biorientations are established, or why heterochromatic homology would be required for this process. The recent discovery of chromatin threads between nonexchange homologs (Hughes *et al.* 2009) and the identification of a phospho-specific antibody that can label threads (Hughes *et al.* 2011) made us consider whether chromatin threads could be detected on chromosomes that undergo HS. Therefore, we did prometaphase preps visualized with anti-pH 3S10 antibodies in both *XXY* females as well as *C(1)/Ø*; *C(4)/Ø* and *C(2L)/Ø*; *C(2R)/Ø* females. One complication in this assay is that when the heterologously segregating chromosomes are out on the spindle, they will likely be located between the *4s* near the spindle poles and the exchange chromosomes near the metaphase I plate. Therefore, it may not be clear whether a thread coming from a heterolog originated there, or is instead associated with the *4* further out on the same spindle arm. However, by looking for threads that do not follow the spindle arc occupied by the *4*s, we were able to find evidence of threads in *Y<=>XX* configurations in *XXY* females (Figure 3A) as well as threads from compound arms in *C(2L)/Ø;C(2R)/Ø* females (Figure 3B).

This confusion with normal *4* threads is not an issue in *C(1)RM*, *y v / Ø*; *C(4)RM*, *ci ey^R^/Ø* females, because there are no free *4s* out toward the poles. When we examined prometaphase I oocytes from this genotype, we had considerable difficulty finding oocytes where the *C(1)* and *C(4)* were separated from the normal autosomes on the spindle far enough to detect threads, with most chromosomes bioriented but positioned too close to the *2* and *3* to allow thread detection, a result consistent with the behavior of the monovalent *C(4)/Ø* shown above. Although such figures were infrequent, we were nevertheless able to identify three with sufficient separation, and in all three figures there was evidence of threads coming from the compound chromosomes (Figure 3C).

## Discussion

Our re-examination of how aberrant chromosome configurations behave in female meiosis has provided several insights into prometaphase I chromosome movements of both normal and aberrant chromosomes. First, these aberrant chromosome configurations complete congression and reach metaphase I arrest with their chromosomes in a single mass. Furthermore, as monovalent *C(1)* and *C(2)* chromosomes can be readily found out on the spindle during prometaphase I, a pairing partner is not necessary for either chromosome movement or congression. Their positioning between the nearby *4* and the exchange chromosomes indicates the monovalents are not moving erratically, implying they have some way to balance their poleward and plateward forces. Although other mechanisms for achieving this balance are possible, one potential mechanism is that there could be heterochromatic threads connecting the monovalent to other paired chromosomes, an idea consistent with the observation of a low rate of *C(1)<=>44* and *C(2)<=>44* configurations. This could be tested in the future by looking for threads connecting monovalent compounds to other chromosomes.

In contrast, prometaphase movement is suppressed in flies with a monovalent *C(4)* chromosome. The *C(4)* chromosome is capable of movement when a normal *4* is also present, but on its own it does so infrequently, even when the *X* is made nonexchange, or when a heterologous *C(1)* pairing partner is available. These results suggest that a possible normal function of the enigmatic dot chromosome seen in many *Drosophila* species may be to facilitate or organize proper chromosome movement on the prometaphase I spindle. A mechanical analogy would be the central pegs securing a tent. The two pegs pull the central guy line of the tent in opposite directions, establishing tension that makes the line rigid enough to support other parts of the tent. However, with only a single peg, tension cannot be established, and the tent structure cannot unfurl. In a normal meiosis, the *4s* move out onto opposite halves of the spindle, remaining connected by a heterochromatin tether. This would make the *4s* analogous to the central pegs, with the thread connecting them as the guy line. 

Because the arc of the spindle between the *4s* has the most intense tubulin staining (Hawley and Theurkauf 1993; Hughes *et al.* 2009), it appears that the presence of free *4s* may alter the structure of the spindle. Little is currently known about how the heterochromatin thread interacts with the spindle, but if threads connecting other chromosomes also can interact with the thicker spindle arc between the *4s*, this may facilitate the normal prometaphase movements of other nonexchange chromosomes. This idea is supported by our finding here that a monovalent *C(4)* is still the chromosome closest to the pole when another nonexchange homologous pair is present, which would not be expected if any nonexchange pair could establish this alteration, and is consistent with the observation that the average *4-4* separation distance during prometaphase shortens when the amount of *4* heterochromatin is reduced (Gilliland *et al.* 2014). We note that the *4* shares heterochromatin sequences with other chromosomes; the AATAT probe used for the *4* also hybridizes to smaller spots on the *X* and *3* (Hughes *et al.* 2009), suggesting the possibility (mentioned previously) that these repeat sequences could form tethers between nonhomologous chromosomes. We plan to test this idea by searching for measurable spindle differences in a *C(4)/Ø* genotype when compared to *4/4* or *C(4)/4*, such as the lack of denser tubulin in the spindle arc containing the *C(4)*.

We must note that our present data cannot rule out several alternative explanations for this lack of chromosome movement onto the prometaphase I spindle, including differences in the overall heterochromatin content of the cell, genetic background differences between strains, or an alternative model where movement is just facilitated by chromosome size, such that if another small chromosome were added to the *C(4)/Ø* background, chromosome movement might be restored. The recently discovered *B* chromosomes of *D. melanogaster* (Bauerly *et al.* 2014) would not be suitable for this latter experiment (as they carry the AATAT repeat and can induce normal *4s* to nondisjoin), but it could be tested by introducing a minichromosome lacking *4* homology into the *C(4)/Ø* background.

Second, the rate that these chromosomes are observed to be oriented at metaphase I arrest is close to the meiotic segregation rates inferred genetically. This confirms the classical genetic inference that the observed segregation pattern is not caused by lethality. Furthermore, these rates also match those from previous studies that counted chromosomes out on the spindle (Xiang and Hawley 2006), which we now know were still in prometaphase I. Because live imaging has demonstrated that chromosomes remain in a stable configuration for several hours during congression (Gilliland *et al.* 2009), this agreement between prometaphase and metaphase rates indicates that heterologous co-orientations are likely established prior to the onset of congression, and those co-orientations are maintained during the slow process of congression, which becomes the most abundant configuration when the oocytes were fixed. We do note that because the *X* and *Y* are both sex chromosomes, and the homology used for segregation in male meiosis may also be used in females, it is possible that the mechanisms that operate during prophase leading to secondary nondisjunction and HS may be different.

Third, we have shown that heterochromatin threads can be found on heterologously segregating chromosomes. This observation is consistent with previous findings that heterochromatic homology can disrupt nonexchange segregation (Hawley *et al.* 1993) and provides a candidate mechanism for how HS are established. Heterochromatic repeats are known to associate with themselves during prophase (Dernburg *et al.* 1996). However, if those chromosomes undergo crossing-over, the heterochromatic associations dissolve (Xiang and Hawley 2006). This finding suggests that heterochromatic homology between any pair of chromosomes could establish connections by this normal intra-repeat association process. Without recombination, the threads would then be in place to establish biorientation during prometaphase I. This suggests both secondary nondisjunction and HS may be mediated by the same mechanism. We note that all heterologous partners tested here have at least some heterochromatic homology (Ferree and Barbash 2009). One prediction of this model is that threads should not be found between chromosomes that completely lack heterochromatic homology. This is a difficult hypothesis to test; even the *C(1)/Ø*; *C(2)/Ø* genotype tested by Dernburg *et al.* (1996), would be predicted to have threads, as the *C(2)* was constructed using *Y* chromosome heterochromatin (Martins *et al.* 2013), and *X-Y* threads can be seen in *XXY* females (Figure 3A). As this stock no longer exists, we made several attempts to recreate it using cold-shock induced nondisjunction (Martins *et al.* 2013), but were unsuccessful. However, as these chromosomes do co-segregate, we would have expected threads to be present. The creation of new compound chromosomes by modern site-specific recombination techniques, as was recently done to construct new ring-*X* chromosomes (Golic and Golic 2010), might be able to address this issue. Indeed, as the genetic contents of these compound chromosomes are often murky (having been constructed through multiple rounds of X-ray rearrangement followed by decades of evolution in stock), such an undertaking might prove broadly useful.

In conclusion, our results demonstrate that heterologous chromosome biorientations are being established during prometaphase I, before the process of congression, and appear to be mediated by similar heterochromatin threads to homologous nonexchange chromosomes. We note here that this process may have been correctly inferred by Grell (1962a) purely from genetic data. She suggested that a round of exchange-mediated pairing occurred first, followed by a second round of pairing for nonexchange and heterologous chromosomes, which she called distributive segregation. The results from *XXY* females demonstrate that if a crossover occurred during prophase, it will take priority for determining the cosegregation of the *Xs*. As recombination occurs much earlier in meiosis than congression, it may be that Grell’s postulated second round of segregation actually corresponds to the process of the chromosomes becoming oriented by the heterochromatic tethers during prometaphase.
